# Clinical efficacy of different marginal forms of endocrowns: study protocol for a randomized controlled trial

**DOI:** 10.1186/s13063-019-3530-1

**Published:** 2019-07-24

**Authors:** Jieli Sun, Wenhao Ruan, Jiahui He, Xiaoyu Lin, Bowen Ci, Siye Yin, Wenjuan Yan

**Affiliations:** Department of Conservative and Endodontic Dentistry, Nanfang Hospital, Southern Medical University, 1838 N Guangzhou Rd, Guangzhou, 510515 China

**Keywords:** Dental defect, Endocrown, Margin, CAD/CAM, Dental restoration, Root canal treatment

## Abstract

**Background:**

After root canal treatment, most tooth defects need to be restored. Onlay restoration is widely used to restore dental defects. Endocrown is a new type of onlay; however, dentists have yet to obtain a full understanding of the clinical effects of marginal forms of endocrowns. Here, we present a multicenter protocol to compare the clinical efficacy of two marginal forms (flat and 90-degree shoulder) for tooth restoration. The efficacy will be evaluated by marginal fit, marginal discoloration, and integrity of restoration.

**Methods:**

The proposed flat and 90-degree shoulder marginal endocrown assessment trial is an open-label, parallel-group, multicenter randomized controlled trial involving two hospitals. A total of 200 patients will be included in this trial, and the following patient inclusion criteria will be applied: good oral hygiene habits, no periodontal diseases, receipt of standard root canal treatment, and need for endocrown restoration. Patients will be enrolled after providing signed informed consent and will be divided into two groups (flat and 90-degree shoulder endocrown) in accordance with a random number table. Treatment allocation will be balanced (1:1). Endocrowns will be cemented by dual-cured luting composite. Clinical evaluations will be performed at baseline and at 24 months after treatment in accordance with modified US Public Health Service criteria by two independent evaluators. The primary outcome will be marginal fit; secondary outcome measures will include debonding, marginal discoloration, and integrity of restoration. All acquired data will be analyzed by an independent statistician. Wilcoxon one-sample tests will be used for intra-group comparisons, and Wilcoxon two-sample tests will be used for inter-group comparisons. The Bonferroni method will be used to correct for multiple comparisons, and hierarchical logistic regression will be applied to determine central effects.

**Discussion:**

The results of this trial will provide a clinical basis for clinicians to restore teeth by endocrowns and to improve long-term restoration for patients.

**Trial registration:**

ClinicalTrials.gov identifier: NCT03398395. Registered on 12 January 2018.

**Electronic supplementary material:**

The online version of this article (10.1186/s13063-019-3530-1) contains supplementary material, which is available to authorized users.

## Background

Pulpal and periapical diseases are the main causes of tooth loss. Root canal treatment (RCT) is the only effective means for treating these diseases. However, in the absence of coronal restoration, failure of RCT is commonly seen. Contamination of the root canal system by saliva, which is referred to as “coronal leakage” or “coronal microleakage”, is a potential cause of endodontic failure [1]. Tooth fracture is another cause. Indeed, teeth are vulnerable to fracture after RCT, the main reasons for which are as follows: (1) Dentin regeneration ceases once secondary dentin and tertiary dentin are interrupted. (2) In some cases, considerable loss of dental tissues has occurred because of deep caries, abrasion, or trauma [2]. (3) Tooth structure is weakened during RCT. As treated teeth are prone to fracture, especially in the coronal region of the posterior teeth, it is very important to restore the teeth within a certain time period after RCT. Moreover, coronal restoration can prevent reinfection of the root canal system or periapical area.

There are many ways to restore teeth after RCT, such as composite resin direct filling, inlay restoration, onlay restoration, full crown restoration, and post-core crown restoration [3]. In onlay restoration, one or more cusps of tooth are covered. This technique not only provides superior aesthetics but also minimizes the loss of healthy tooth tissue, making it an attractive treatment choice for posterior teeth with extensive cavities due to caries [4]. Moreover, by covering more than one tooth cusp, onlay provides a favorable distribution of stress, reducing the fracture risk of tooth or restoration or both. Ceramic onlay restoration showed promising results, yielding a 92.5% success rate, in a 4-year investigation of extensively restored, endodontically treated molars [5]. These observations suggest that onlay restorations are an optimal approach to restoring posterior teeth after RCT.

Endocrown is a new type of onlay with a retainer in the pulp cavity [6, 7]. It consists of a cervical margin in the form of a butt joint and a preparation of the pulp chamber. The endurance and stability of the restoration derive from the adhesion of cementation and increased stress sharing as well as the interface provided by the pulp cavity retainer [8]. This restoration method is not only effective for preserving residual tooth tissue but also suitable for severely damaged molars or premolars after dental pulp treatment [9]. Compared with post-core crown techniques, endocrown restoration is simpler because of the core-crown integrity; furthermore, no post is needed, reducing the risk of root fracture [10]. Post-core crown restoration is aimed at strengthening residual dental hard tissue and replacing missing dental tissue. The resistance of the tooth cervix is not enhanced, and the post only provides the retaining force for the crown [11]. Furthermore, post-core crown restoration can present additional risks, such as canal perforation and root fracture. Full crown restoration is also widely performed. A finite element analysis of a full crown and endocrown revealed that the endocrown was superior to the traditional full crown [12]. According to biomechanics, the strength of the dental tissue depends on the amount of dental hard tissue and its internal strength and anatomical characteristics. The characteristics of dental tissue are affected by caries, the preparation of the cavity, the path of access to the canals, and the enlargement of the root canal. However, Dietschi et al. showed that the effects of endodontic treatment on dental biomechanical properties are limited [13]. A three-dimensional finite element study showed that the stress distribution on enamel, dentin, and adhesive surface was smaller for endocrown than for all-porcelain onlay and traditional full crown [14, 15]. In addition, several experiments showed that endocrown had greater fracture resistance than traditional completed crown [14–16]. A retrospective study of endocrown restorations reported a clinical success rate of 94–100% [17] and demonstrated that the retention effect of endocrown restoration was superior to that of traditional full crown restoration. Endocrown restoration can preserve residual tooth tissue to a greater extent than can other restoration methods. It is especially useful for teeth with a low occlusogingival distance that have insufficient retention ability for a full crown.

**Fig. 1:**
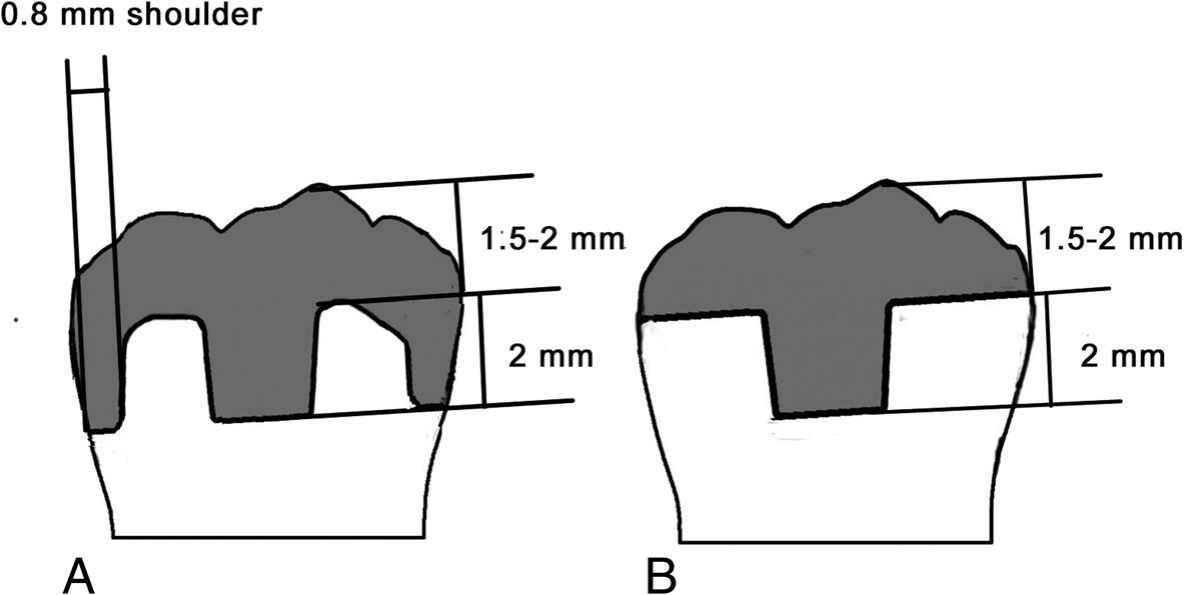
Schematic representations of the two marginal forms of endocrown. A. Ninety-degree shoulder endocrown (intervention group). B. Flat endocrown (control group).

Studies have shown that the type of marginal form has a significant effect on the prognosis of prostheses after RCT and restoration with full crowns [18–20]. The study by Taha et al. showed that adding a 1-mm shoulder finish line can increase the endocrown’s fracture resistance. However, further investigations, especially on fatigue behavior, are needed [21]. The restoration ability of endocrowns is affected by the bonding surface and the amount of residual tooth tissue [22]. The enamel layer distribution of the teeth bases, which determines the composition of the bonding surface as well as the residual amount of dental tissue, varies according to the preparation of the different marginal forms of endocrowns. A flat endocrown shows more residual tooth tissue and less bonding area, whereas a 90-degree shoulder endocrown leads to a larger bonding area and less residual tooth tissue. Whether there is a significant difference in restorative effect between these two forms is difficult to predict. Therefore, two common marginal forms of endocrowns will be designed in this study to observe their restoration effects (Fig. 1).

CAD/CAM is a type of computer-aided design and manufacturing software. The dental CAD/CAM system is used to collect imaging data of digital impressions through a built-in camera and does not require the intermediate links of traditional restoration systems, such as preparing molds and fillings. Therefore, this system can avoid possible errors during milling with numerical control machine tools. Because of its advantages in precision and clinical restoration, it is increasingly favored by patients and dentists [23, 24]. In recent studies, the system was used to successfully prepare complex prostheses that cannot be produced by traditional methods and was found to significantly shorten the production time of restorations [25].

Along with the rapid innovations in digital dentistry, additional chair-side CAD/CAM materials have been developed. According to the latest classification system, all-ceramic and ceramic-like restorative materials can be categorized into three groups, depending on the phase or phases present in their chemical composition: (1) glass-matrix ceramics, (2) polycrystalline ceramics, and (3) resin-matrix ceramics. All three types offer good aesthetic performance, mechanical properties, and biocompatibility [26, 27]. Glass-matrix ceramics are non-metallic inorganic ceramic materials that contain a glass phase and a dispersed crystalline phase (crystals) and are divided into three subgroups: feldspathic ceramics, synthetic ceramics, and glass-infiltrated ceramics. Synthetic ceramics can effectively prevent the expansion of cracks and improve the strength and cutting performance of the materials because of the greater presence of the crystalline phase than of the glass phase. Leucite-reinforced lithium disilicate and zirconia-reinforced lithium silicate ceramics are representatives of synthetic glass ceramics. Lithium disilicate ceramic (IPS e.max CAD, Ivoclar Vivadent, Amherst, NY, USA) is composed of about 70 vol.% of crystalline phase incorporated in the glassy matrix. The intermediate crystalline phase or “blue” state has 130 ± 30 MPa flexural strength, and the final flexural strength can reach 360 ± 60 MPa. It is widely used in clinical practice because of its good flexural strength and shade; however, it is also disadvantageous because the 15–20% contraction of the material after polymerization may lead to a reduction in the density between the onlay and teeth during the second sintering process [28]. Zirconia-enhanced lithium silicate glass ceramic (VITA SUPRINITY, VITA Zahnfabrik, Bad Säckingen, Germany) contains lithia (15–21 wt.%) and zirconia (8–12 wt.%) (about 10 times more zirconium dioxide than in traditional CAD/CAM glass ceramic). Its flexural strength can reach 450 MPa after glazing. Its ultrafine (0.5-μm) granules guarantee a homogeneous material structure to avoid polymerization shrinkage during the second sintering step. In addition, it has good emulsion fluorescence, which ensures an improved clinical polishing effect [29]. Experimental studies showed that VITA SUPRINITY had greater fracture toughness, flexural strength, elastic modulus, and hardness than disilicate glass ceramics.

Despite its advantages, VITA SUPRINITY has seldom been used in the design of different marginal forms of endocrowns. Therefore, we will use it as the experimental material in this study based on the dental CAD/CAM system.

## Methods and design

### Hypothesis

For this study, we hypothesize that the restorative effect of the 90-degree shoulder endocrown will be superior to that of the flat endocrown restoration.

### Objective

The objective is to compare differences between the two marginal forms of endocrowns (i.e., 90-degree shoulder and flat) by assessing the firmness, comfort, and durability of dental restorations after RCT using VITA SUPRINITY blocks designed and manufactured using a chair-side CEREC CAD/CAM system (Dentsply Sirona, Bensheim, Germany).

### Methods

**Fig. 2:**
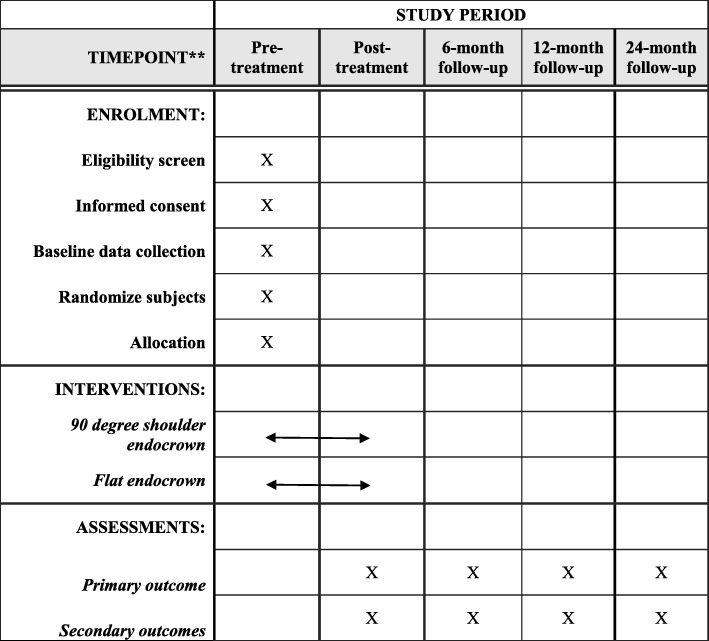
SPIRIT (Standard Protocol Items: Recommendations for Interventional Trials) Figure

This is a multicenter, randomized, open-label superiority trial with two balanced parallel arms. The participants will be recruited from the Departments of Conservative and Endodontic Dentistry in Nanfang Hospital of Southern Medical University and the Guanghua School of Stomatology of Sun Yat-sen University. The schedule of enrollment, interventions and assessments is showed in Fig 2.

### Inclusion criteria

1. The patient is healthy and is 18–60 years old and has molar teeth and tooth root apex without evident damage and no root fracture, as determined by x-ray.
2. The patient has three or four walls of intact tooth tissue after complete root canal therapy.
3. The patient has good oral hygiene.
4. The patient has signed an informed consent form.
5. The patient is not participating in any other clinical trial.
6. The patient has received a class A assessment according to the modified US Public Health Service (USPHS) criteria for marginal adaptation after restoration.

### Exclusion criteria

1. Obvious destruction of the apical tissue or presence of large cysts or both
2. Severe periodontitis
3. Oral malignant tumor(s)
4. Undergoing radiotherapy
5. Pregnancy
6. Mental illness or systemic diseases
7. Incapable of self-care
8. Unsuitable for the trial as deemed by the researchers

### Dropout criteria

1. Poor clinical compliance
2. Voluntary withdrawal from the trial by the patient

According to the software nQuery Advisor + nTerim 3.0, a sample size of 160 patients (80 for each group) should be recruited [30]. To allow for 20% attrition, the recruitment sample size for this trial will be 200 patients (100 in each group).

Upon fulfilment of the selection criteria, 200 eligible participants will be randomly divided into intervention and control groups in a 1:1 allocation ratio. Randomization will be performed in accordance with a random list of numbers generated by the Department of Biomedical Statistics of Southern Medical University. Five dentists will participate in this study and all of them will have received standardized training in endocrown restoration before the study begins. The number of cases assigned to each dentist is unknown and will vary because of factors outside the control of the study. These five dentists will not be involved in observations or data collection during the clinical evaluations.

### The intervention group

The intervention group will receive 90-degree shoulder endocrown restoration. The restoration will be completed by the following steps: (1) All decayed or damaged areas of the tooth will be removed, and occlusal anatomical reduction of 2.0 mm will be performed to form a 90-degree rounded shoulder margin. Gutta-percha will be removed to a depth not exceeding 2 mm and sealed with composite resin. The undercuts of the tooth cavity will be blocked out with a nano-hybrid composite resin, which will serve as a base material. A 2- to 5-degree divergence of the vertical walls will be prepared with a conical flat-end diamond bur. (2) A chair-side CEREC CAD/CAM system will be used to mill VITA SUPRINITY blocks to obtain the prosthesis. (3) Cementation, occlusion adjustment, and polishing will be performed. (4) Patients whose affected tooth receives a class A assessment according to the modified USPHS criteria of marginal adaptation will be included in this trial.

### The control group

The control group will be treated with flat endocrown restoration. In the first step of the restoration, all of the decayed or damaged areas of the tooth will be removed, and occlusal flat reduction of 1.5–2.0 mm will be achieved. The subsequent steps will be the same as those for the intervention group.

### Outcome measures

Clinical evaluations will be performed at baseline and at 24 months after treatment according to modified USPHS criteria by two independent evaluators (Table 1) [31–34]. The evaluators will complete a standardized training program before the experiment begins. If two evaluators present inconsistent evaluations during the study, a third evaluator will perform an evaluation, and the concurring evaluations from two evaluators will be used for analysis.

**Table 1:** Modified USPHS criteria

Category	Rating	Criteria
Marginal adaptation	A	Probe, does not catch, smooth margin interface
	B	Probe catches at single spots, slight roughness
	C	Probe catches under 50% of margin length
	D	Probe catches at/over 50% of margin length
Marginal discoloration	A	No discoloration on the margin
	B	Superficial discoloration on the margin, no penetrate in pulp direction
	C	Discoloration has penetrated the margin in pulp direction
Integrity of restoration	A	Completely intact
	B	Crack apparent on transillumination
	C	Fracture observable
	D	Crown lost(state at which interface debond occurred)

### Data collection

The investigators will use a case report form (CRF) to collect data for the outcome analysis. The CRF includes demographic data, oral habits, medical history, and adverse events. To protect the privacy of patients, the patients will be registered with the first letters of their full name on the form. A clinical researcher will visit each center to inspect the acquired data and assess data quality by comparing the data with the medical records. The data will be entered twice into a database by designated operators and inspected by a data manager.

### Statistical methods

#### Basic principles

The data will be analyzed by an independent statistician. All statistical tests will be two-tailed. A *P* value of less than 0.05 will be the level of significance, and 95% confidence intervals will be calculated. Parametric methods will be considered first. Data that do not meet or cannot be transformed to meet parametric assumptions will be analyzed by non-parametric methods.

#### Primary outcome analysis

Wilcoxon one-sample tests will be used for intra-group comparisons, and Wilcoxon two-sample tests will be used for inter-group comparisons. The Bonferroni method will be used for multiple comparisons, and hierarchical logistic regression will be used to detect central effects.

#### Secondary outcome analysis

For intra-group comparisons, paired *t* tests or Wilcoxon one-sample tests will be used for quantitative variables, and McNemar tests will be used for qualitative variables. For inter-group comparisons, quantitative variables will be analyzed by one-way analysis of variance (more than two groups) and two-sample *t* tests (two groups) or by non-parametric methods. Qualitative variables will be analyzed by Pearson’s chi-squared tests. Non-parametric data will be analyzed by the Kruskal–Wallis test.

## Discussion

This trial will help clinicians provide their clients with evidence-based options regarding marginal forms of endocrowns. However, owing to limitations associated with dental defects after RCT, the 90-degree shoulder endocrown is not suitable for all tooth restorations. Therefore, the selection of marginal forms should be based on the conditions of the dental defects.

This randomized clinical trial (see SPIRIT check list the Additional file 1) may lead to an improvement in the survival rate of restorations for patients. For researchers, it may provide input for further research concerning the marginal forms of endocrowns.

### Trial status

This trial is in the process of recruiting participants.

## Additional file

Additional file 1: SPIRIT (Standard Protocol Items: Recommendations for Interventional Trials) 2013 Checklist: Recommended items to address in a clinical trial protocol and related documents*. (DOC 135 kb)

## Data Availability

All data are fully available without restriction.

## Abbreviations

CAD/CAM: Computer-aided design/computer-aided manufacture
CRF: Case report form
RCT: Root canal treatment
USPHS: US Public Health Service
