# Supplementary material for: Clinical efficacy of different marginal forms of endocrowns: study protocol for a randomized controlled trial
Source: Trials. 2019 Jul 24;20:454. doi: 10.1186/s13063-019-3530-1 (PMC6657076; doi:10.1186/s13063-019-3530-1)
Supplement: Supplementary file 1 — SPIRIT (Standard Protocol Items: Recommendations for Interventional Trials) 2013 Checklist: Recommended items to address in a clinical trial protocol and related documents*. (DOC 135 kb) [file 13063_2019_3530_MOESM1_ESM.doc]

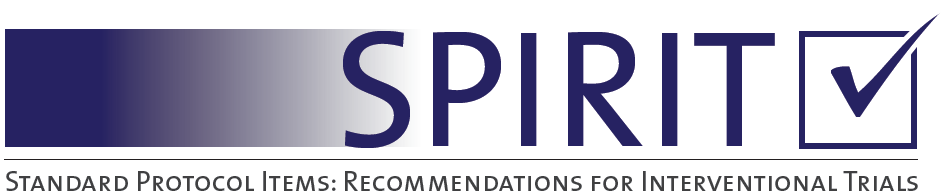


SPIRIT 2013 Checklist: Recommended items to address in a clinical trial protocol and related documents*

| Section/item | ItemNo | Description |
| --- | --- | --- |
| **Administrative information** | | |
| Title | 1 | Clinical efficacy of different marginal forms of endocrown: study protocol for a randomized controlled trial |
| Trial registration | 2a | Protocol ID: NFEC-2017-141; ClinicalTrials.gov ID: NCT03398395; Title: Endocrown and 90°shoulder Endocrown |
|  | 2b | Table 1. |
| Protocol version | 3 | Date: 2018-01-19; Author: Wenjuan Yan |
| Funding | 4 | This trial is funded by a grant from the Southern Medical University, China (LC2016PY023). |
| Roles and responsibilities | 5a | Wenjuan Yan developed and improved the trial design. Jieli Sun drafted the manuscript, which was carefully revised, edited. Jiahui He and Wenhao Ruan conducted patient follow-up investigation. Xiaoyu Lin and Bowen Ci recruited participants. Siye Yin carried out patient care cooperation work. |
|  | 5b | Trial sponsor: Wenjuan Yan, E-mail: 645613053@qq.com |
|  | 5c | This funding source had no role in the design of this study and will not have any role during its execution, analyses, interpretation of the data, or decision to submit results |
|  | 5d | Data management team from the Department of Biomedical Statistics, Southern Medical University. |
| Introduction |  |  |
| Background and rationale | 6a | After root canal treatment, most tooth defects need to be restored. Onlay restoration is widely used in the restoration of dental defects. Endocrown is a new type of onlay, but as for its marginal forms dentists have come to no unified understanding clinically. Here, we present a multicenter protocol to compare two marginal forms (flat and 90 degree shoulder) for tooth restoration in terms of the clinical efficacy from the perspective of marginal adaptation, marginal discoloration, and integrity of restoration. |
|  | 6b | The restoration effect of endocrown is affected by the bonding surface and the amount of residual tooth tissue. The enamel layer distribution of the base teeth, which determines the composition of the bonding surface, as well as the residual amount of dental tissue, are various according to the preparation of different marginal forms. Flat endocrown shows more residual tooth tissue and less bonding area while 90 degree shoulder endocrown leads to larger bongding area and less residual tooth tissue. Whether there is significant difference in restoration effect among these two forms is difficult to predict. Therefore, two common marginal forms of edge were designed in this study to observe the restoration effect. |
| Objectives | 7 | Specific objectives Find the best way to restore the tooth defect  Hypotheses: the restoration effect of the participants with 90 degree shoulder endocrown is better than the participants with flat endocrown. |
| Trial design | 8 | This trial is a multicenter, randomized, open-label superiority trial with two balanced parallel arms. |
| Methods: Participants, interventions, and outcomes | | |
| Study setting | 9 | Academic hospital: Nanfang Hospital, Southern Medical University, Guangzhou, 510515, China |
| Eligibility criteria | 10 | Inclusion criteria  1) Healthy patients aged 18-60 years with molar teeth, tip root without evident damage and no root fracture by X-ray;  2) 3 or 4 walls of intact tooth tissues after complete root canal therapy;  3) Good oral habits;  4) Having signed informed consent form;  5) Excluding from other clinical trials;  6) Assessment as Class A in line with the modified USPHS criteria for marginal adaptation after restoration.  Exclusion Criteria  1) Obvious destruction of the apical tissue and large cysts;  2) Severe periodontitis;  3) Oral malignant tumor;  4) Undergoing radiotherapy;  5) Pregnant women;  6) Mental illness or systemic diseases;  7) Patients who are not self-cared;  8) Other patients unsuitable for the trials in the researchers’ view. |
| Interventions | 11a | Upon fulfillment of selection criteria, 200 eligible participants will be randomly allocated into the intervention and control groups in a 1:1 allocation ratio. The patients in the intervention group will be allocated to be restored by 90 degree shoulder endocrown. Patients in the control group will be allocated to be restored by the flat endocrown. |
|  | 11b | Study suspension: Poor clinical compliance; Quitting the RCT voluntarily; |
|  | 11c | Each researcher will be given formal training before the study begins.As the study progresses, patient information will be entered into a computer and not seen by people outside the trial |
|  | 11d | Physicians are prohibited from failing to follow a random list of interventions. |
| Outcomes | 12 | The primary outcome, clinical efficacy of restoration, will be measured upon the modified USPHS criteria. When a case is evaluated with all the three items at level A, the restoration is considered as a success. When a case has one item at level B and the other two at the level no lower than level B, the restoration is considered as acceptable, requiring further observation. If a case has any item at level C or level D, it is considered as a failure. Secondary outcomes will include marginal adaptation, marginal discoloration and integrity of restoration. Survival may be evaluated if the follow-up is extended. |
| Participant timeline | 13 | Fig.1 |
| Sample size | 14 | In this procedure, we used the formula for calculating the sample size, and a sample size of 160 patients (80 for each group) should be recruited. Allowing 20% attrition, the recruited sample size for this trial will be of 200 patients (100 in each group). |
| Recruitment | 15 | Each clinical center involved in the study was chosen based on documentation for patient availability, among other things. It is, however, worthy to note the specific plans of each center.  1. Tooth defect patient Lists;  2. Advertisements;  3. Give each participant an appropriate road subsidy and free oral examination. |
| **Methods: Assignment of interventions (for controlled trials)** | | |
| Allocation: |  |  |
| Sequence generation | 16a | Upon fulfillment of selection criteria, 200 eligible participants will be randomly allocated into the intervention and control groups in a 1:1 allocation ratio. Randomization will be performed according to a random list of numbers generated by the Department of Biomedical Statistics, Southern Medical University. |
| Implementation | 16b | Participants will be randomised. |
| Implementation | 16c | Randomization  All patients who give consent for participation and who fulfil the inclusion criteria will be randomized. Randomisation will be requested by the staff member responsible for recruitment and clinical interviews from CenTrial [Coordination Centre of Clinical Trials]. In return, CenTrial will send an answer form to the study therapist who is not involved in assessing outcome of the study. This form will include a randomisation number. In every centre closed envelopes with printed randomisation numbers on it are available. For every randomisation number the corresponding code for the therapy group of the randomisation list will be found inside the envelopes. The therapist will open the envelope and will find the treatment condition to be conducted in this patient. The therapist then gives the information about treatment allocation to the patient. Staff responsible for ecruitment and symptom ratings is not allowed to receive information about the group allocation.  The allocation sequence will be generated by the Department of Biomedical Statistics, Southern Medical University. Throughout the study, the randomisation will be conducted by centre. The randomisation list remains with Centre for the whole duration of the study. Thus, randomisation will be conducted without any influence of the principal investigators, raters or therapists. |
| Blinding (masking) | 17a | This trial is an open trial. |
|  | 17b | This trial is an open trial. |
| **Methods: Data collection, management, and analysis** | | |
| Data collection methods | 18 | Clinical reevaluations will be separately performed at baseline and 24 months after insertion according to the modified USPHS criteria by 2 independent evaluators (Table1) [22-26]. The evaluator will conduct a standardized training program before the experiment begins. If two evaluators have different evaluations during study, a third evaluator will be involved. And the final data will use the same opinion of two evaluators.  The investigators will use a Case Report Form (CRF）to collect the data for outcomes analysis. The CRF covered demographic data, oral habits, history and adverse events. To protect the privacy of patients, the patients will be registered with their first letters of their full name at filling the form. A clinical researcher will visit each center to check the acquired data and assess the data quality by comparing them with the medical records. The data will be entered twice into the database by designated operators and checked by a data manager. |
| Data management | 19 | Double data entry |
| Statistical methods | 20a | SAS9.4 statistical software was used for statistical analysis. |
|  | 20b | The matching t test or the corresponding non-parametric method are used for intra-group comparison. The covariance analysis was used to compare the groups. The baseline was taken as the covariable and the central effect and the possible interaction effect were considered. Multiple comparisons were performed using the LSD method. |
|  | 20c | Missing data: The last observation carry forward method was adopted to fill the validity analysis, that is, the case data that failed to observe the whole treatment process were transferred to the final result of the test with the last observation data.The security evaluation does not evaluate missing data. |
| **Methods: Monitoring** | | |
| Data monitoring | 21a | DMC consists of the Department of Biomedical Statistics, Southern Medical University, and it mainly responsible for data management and statistical analysis. It is independent from the sponsor and competing interests. |
|  | 21b | No interim analysis was performed in this study. |
| Harms | 22 | In our study an adverse event will be defined as any untoward medical occurrence in a subject without regard to the possibility of a causal relationship. The adverse events include the materials in the restoration process are allergic, and the prosthesis falls off, leading to aspiration and aspiration. Any serious adverse events occurring in the course of the test shall be reported to the medical ethics committee of the unit and the applicant immediately, and the "report form of serious adverse events" shall be filled in. If it is a serious adverse reaction, it shall also be reported to the state drug supervision and administration within 24 hours. |
| Auditing | 23 | The frequency of audit is once a year. The project organization will review the test process and make comprehensive evaluation. Eliminate funding for lower ranked projects. The process will be independent from investigators and the sponsor. |
| Ethics and dissemination | | |
| Research ethics approval | 24 | The trial has been approved by the Medical Ethics Committee of Nanfang Hospital, Southern Medical University. This protocol has been reviewed and approved by the sponsor and the ethical committees. Subsequent to initial review and approval, the sponsor and the ethical committees will review the protocol at least annually. The Investigator will make safety and progress reports to the ethical committees at least annually and within three months of study termination or completion at her site. |
| Protocol amendments | 25 | Any amendments to the protocol will be reviewed and approved by the ethics committee and funding support departments. |
| Consent or assent | 26a | Trained Researchers will introduce the trial to patients. Patients will also receive information sheets. Researchers will discuss the trial with patients. Researchers will obtain written consent from patients willing to participate in the trial. |
|  | 26b | Not applicable |
| Confidentiality | 27 | All study-related information will be stored securely at the computer. All participant information will be stored in locked file cabinets. All reports, data collection, process, and administrative forms will be identified by a coded ID number. |
| Declaration of interests | 28 | All academic conferences and research activities related to this study will be reimbursed. |
| Access to data | 29 | The Data Management Coordinating Center will oversee the intra-study data sharing process, with input from the Data Management Subcommittee. All Principal Investigators will be given access to the data. All data sets will be password protected. Project Principal Investigators will have direct access to their own site’s data sets, and will have access to other sites data by request. To ensure confidentiality, data dispersed to project team members will be blinded of any identifying participant information. |
| Ancillary and post-trial care | 30 | Patients who participated in the study could receive compensation from the study unit, including additional medical care, compensation or damages. |
| Dissemination policy | 31a | After the completion of the experiment, the researcher submitted the experimental results to the ethics review, and then published the results data paper. |
|  | 31b | All the researchers involved in the experiment can be the authors of the paper and enjoy the research results of this study. |
|  | 31c | After 3 years, we will provide complete data sets to the appropriate data files for sharing. |
| Appendices |  |  |
| Informed consent materials | 32 | Appendix: Informed Consent |
| Biological specimens | 33 | No plans for collection, laboratory evaluation, and storage of biological specimens for genetic or molecular analysis in the current trial and for future use in ancillary studies. |

*It is strongly recommended that this checklist be read in conjunction with the SPIRIT 2013 Explanation & Elaboration for important clarification on the items. Amendments to the protocol should be tracked and dated. The SPIRIT checklist is copyrighted by the SPIRIT Group under the Creative Commons “[Attribution-NonCommercial-NoDerivs 3.0 Unported](http://www.creativecommons.org/licenses/by-nc-nd/3.0/)” license.
